# Association Between Tibial Plateau Fractures and Bone Metabolic Status: A Prospective Observational Study

**DOI:** 10.7759/cureus.92476

**Published:** 2025-09-16

**Authors:** Constantinos D Apostolou, Vassiliki Markopoulou, Georgios Chatzipanagiotou, Stefanos Efstathiou, Efstathios Chronopoulos

**Affiliations:** 1 Orthopaedics and Trauma, Evangelismos General Hospital, Athens, GRC; 2 Orthopaedic Surgery, National and Kapodistrian University of Athens, Athens, GRC; 3 Orthopaedic Surgery, Konstantopoulio General Hospital, Nea Ionia, Athens, GRC

**Keywords:** bone density scan, dual-energy x-ray absorptiometry (dexa), lower extremity trauma, osteoporosis, tibial plateau fractures, vitamin d, vitamin d levels (vit d level)

## Abstract

Background: Tibial plateau fractures are serious injuries affecting knee function and mobility. While typically associated with high-energy trauma, these fractures are increasingly observed in low-energy mechanisms, particularly among patients with compromised bone health.

Objective: To investigate the relationship between tibial plateau fractures and bone health parameters, specifically focusing on vitamin D status and bone mineral density, and to determine if fracture classification correlates with metabolic bone disease indicators.

Methods: A prospective observational study was conducted over 11 months at Evangelismos General Hospital in Athens, Greece. A total of 45 patients with tibial plateau fractures and 28 control patients with knee contusions were assessed. All participants underwent biochemical testing and dual-energy X-ray absorptiometry (DEXA) scanning. Fractures were divided using the Schatzker classification.

Results: A total of 34 (75.6%) patients with tibial plateau fractures had vitamin D deficiency, and 14 (31.1%) were diagnosed with osteopenia. Osteopenia was significantly more prevalent in low-energy trauma patients (six among nine patients vs. eight among 36 patients in high-energy trauma; 66.7% vs. 22.2%, p = 0.017). However, no statistically significant correlation was found between fracture severity and metabolic markers.

Conclusion: A substantial proportion of patients with tibial plateau fractures exhibit suboptimal bone health, especially vitamin D deficiency and osteopenia. Routine metabolic screening is recommended, particularly for fractures caused by low-energy mechanisms.

## Introduction

Tibial plateau fractures represent complex injuries of the proximal tibia involving the articular surface of the knee, and account for approximately 1% of all fractures in the general population [1]. These injuries are of particular clinical interest due to their potential to cause long-term functional impairment, post-traumatic arthritis, and gait instability [2]. While high-energy trauma remains the most common cause of such fractures, particularly in younger individuals involved in motor vehicle accidents or sports injuries [3], there is an increasing incidence of tibial plateau fractures among elderly patients resulting from low-energy mechanisms, such as simple falls.

The rising prevalence of low-energy tibial plateau fractures in older adults highlights the role of compromised bone quality, particularly osteopenia and osteoporosis, in the pathogenesis of such injuries [4]. Osteoporosis is a systemic skeletal disease characterized by low bone mass and microarchitectural deterioration, leading to increased bone fragility and fracture risk [5]. It may be classified as primary, related to aging or postmenopausal estrogen deficiency, or secondary, resulting from underlying medical conditions or pharmacologic treatments such as corticosteroids, hypogonadism, malabsorption syndromes, and endocrine disorders [6-10]. In particular, vitamin D deficiency has been strongly implicated in the pathogenesis of osteoporosis and impaired bone healing, as it compromises calcium homeostasis and bone remodeling [11].

Bone mineral density (BMD), measured by dual-energy X-ray absorptiometry (DEXA), remains the gold standard for diagnosing osteopenia and osteoporosis. According to the World Health Organization, a T-score ≤ -2.5 defines osteoporosis, while values between -1.0 and -2.5 define osteopenia [12]. Several studies have suggested that individuals with low BMD are at higher risk of sustaining periarticular fractures, including those of the tibial plateau [13]. Despite these findings, the relationship between tibial plateau fracture patterns, classified radiographically using Schatzker classification [14], and underlying bone quality remains poorly defined in the literature. Furthermore, the contribution of metabolic factors, such as serum 25-hydroxy vitamin D, calcium, and phosphate levels, to fracture risk and healing in these patients has not been fully elucidated.

The purpose of this prospective study was to investigate whether tibial plateau fractures, as classified by Schatzker, are influenced by bone quality parameters, including the presence of osteopenia or biochemical markers of metabolic bone disease. Additionally, we aim to assess the prevalence of vitamin D deficiency and related metabolic disturbances in patients presenting with such fractures, to explore potential implications for treatment planning and secondary fracture prevention, and to evaluate the necessity of standard endocrinological input.

## Materials and methods

This prospective observational study was conducted over an 11-month period, from March 2024 to February 2025, at Evangelismos General Hospital in Athens, Greece, the largest tertiary referral hospital in the country. The study protocol received approval from the hospital's ethics committee, and informed consent was obtained from all participants prior to their inclusion.

A total of 73 patients were included in the study. Of these, 45 patients were diagnosed with tibial plateau fractures and comprised the study group, while 28 patients who presented with knee contusions, but no fractures, served as the control group. Our rational choice of the control group was to choose among patients with no fracture but with a common orthopedic problem in the same area studied. All enrolled patients were adults (aged ≥18 years) and were evaluated upon admission.

For all participants, clinical data were collected, including age, sex, mechanism of injury, and whether the trauma was classified as high-energy or low-energy trauma. High-energy trauma was defined as a fall from a height greater than 3 meters or a traffic accident at a speed above 30 km/h. Low-energy trauma was considered any injury from standing height or lower.

All fracture patients underwent routine radiographic imaging (X-rays and CT scans) to confirm diagnosis and classify the fracture using the Schatzker classification. Laboratory tests were performed on the day of hospital admission and included measurements of serum calcium, serum phosphate, alkaline phosphatase (ALP), 25-hydroxyvitamin D (25(OH)D), C-reactive protein (CRP), and erythrocyte sedimentation rate (ESR). In addition, all participants underwent BMD assessment via DEXA within a few days after hospital discharge.

Statistical analysis

The values of quantitative variables were presented using the mean ± standard deviation or median with the interquartile range, while for qualitative variables, frequencies (n) and percentages (%) were used. The normality of the data distribution was tested using the Shapiro-Wilk test. Comparison of vitamin D between the groups (plateau vs. control) was conducted using the independent samples median test, as the data did not follow a normal distribution. The predictive value of the biochemical markers in distinguishing plateau fractures from controls was evaluated using receiver operating characteristic (ROC) analysis by calculating the area under the curve (AUC). A multiple logistic regression model was used to assess the independent effect of clinical and biochemical markers on the occurrence of plateau fractures. The association between categorical variables was examined using Fisher’s exact test. All statistical analyses were performed using the SPSS statistical software package version 21.00 (IBM Corp., Armonk, NY). All tests were two-sided, and a p-value < 0.05 was considered statistically significant.

## Results

A total of 73 individuals participated in the study, including 45 patients with tibial plateau fractures and 28 controls. Among the fracture group, 33 (73.3%) patients were male and 12 (26.7%) were female. A total of 38 (84.4%) patients were younger than 63 years, while seven (15.6%) were aged 63 years or older, and the mean age of the group was 49 years. According to Schatzker classification, 18 patients (40%) presented with type I-III fractures (lateral plateau), and 27 (60%) had type IV-VI fractures (medial or bicondylar). Among the control group, 20 individuals (71.4%) were male and eight (28.6%) were female, with the mean age of the group being 34 years. The two groups were homogeneous in terms of gender (p = 1.000) but not in terms of age (p < 0.005), with the control group consisting of younger individuals.

**Figure 1:**
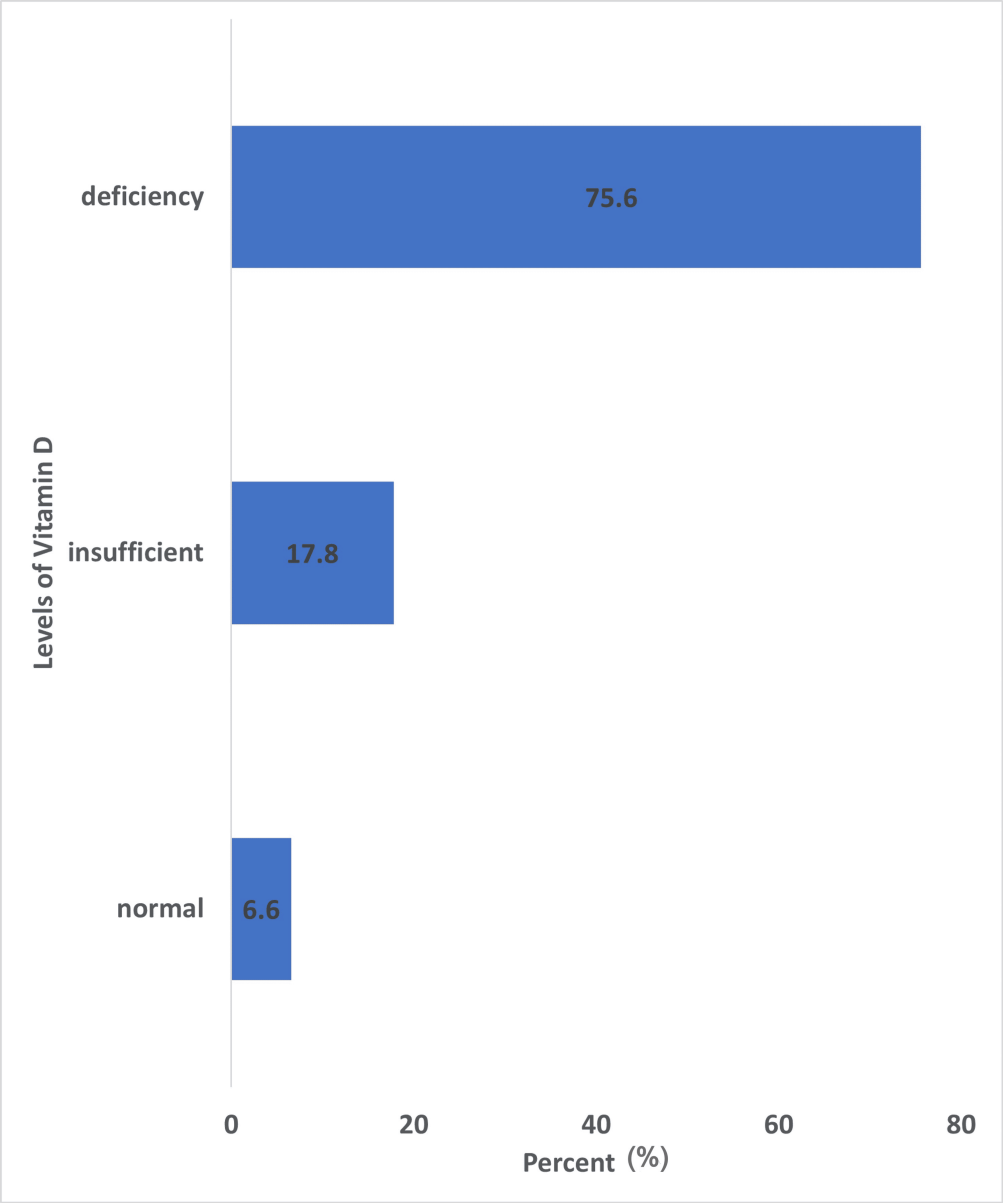
Vitamin D level in patients with tibial plateau fractures.

**Figure 2:**
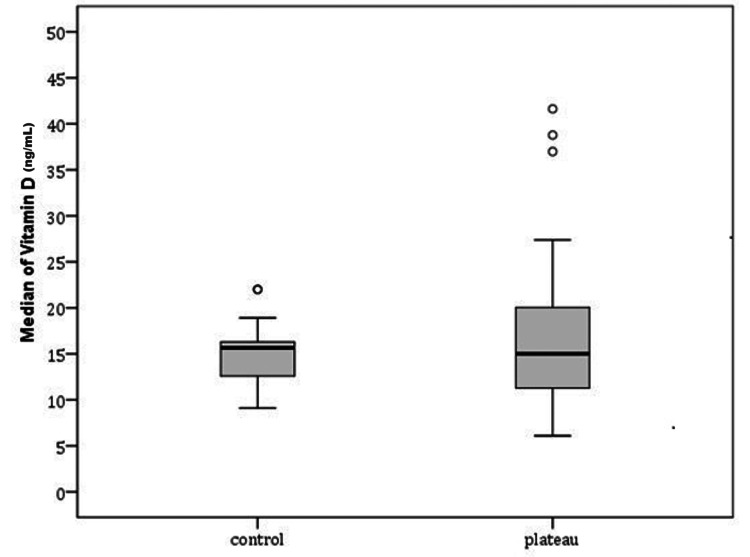
Comparison of vitamin D between groups.

**Figure 3:**
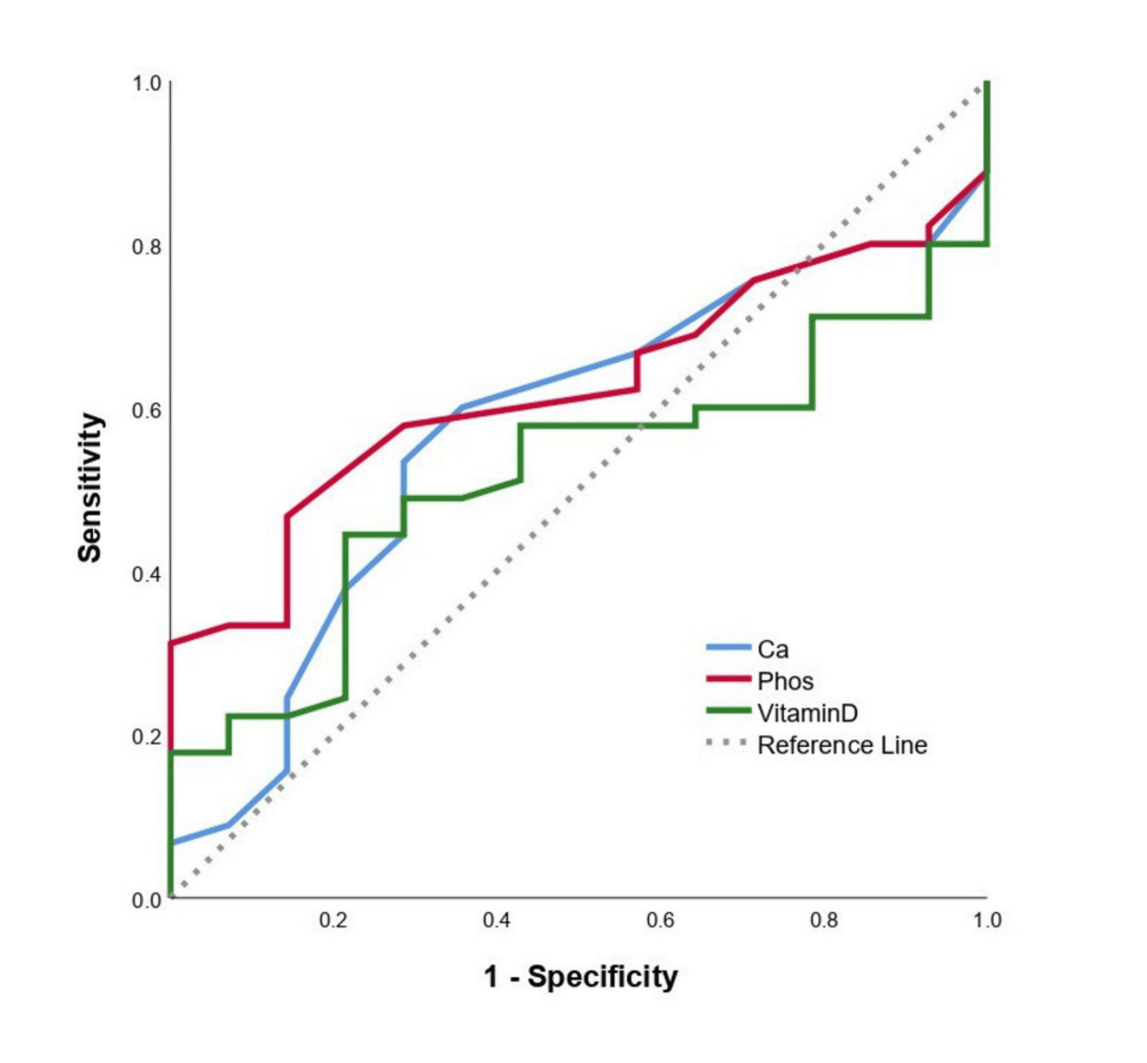
ROC analysis of biochemical markers in the differentiation between plateau fracture and control. ROC: receiver operating characteristic; Ca: calcium; Phos: phosphate.

The majority of tibial plateau fractures (36, 80%) resulted from high-energy trauma, while nine (20%) occurred following low-energy mechanisms. A significantly higher percentage of men sustained high-energy injuries (30 among 33) compared to women (six among 12) (90.9% vs. 50%, p = 0.006). Evaluation of BMD revealed that 14 (31.1%) patients with tibial plateau fractures had osteopenia, and 31 (68.9%) had normal values, whereas all individuals in the control group (n = 28) had normal values of BMD (Table 1). No cases of osteoporosis were recorded in either the study or control group. Women showed a significantly higher prevalence of osteopenia (10 among 12) compared to men (four among 33) (83.3% vs. 12.1%, p < 0.005). There was no significant difference in the prevalence of osteopenia between patients with Schatzker type I-III (six among 18) and those with type IV-VI fractures (eight among 27) (33.3% vs. 29.6%, p = 1.000). Furthermore, patients who sustained fractures due to low-energy trauma had a significantly higher prevalence of osteopenia (six among nine) than those with high-energy trauma (eight among 36) (66.7% vs. 22.2%, p = 0.017). The median serum 25(OH)D levels in the fracture and control groups were 15 ng/mL and 15.63 ng/mL, respectively. Among the patients, 34 (75.6%) were classified as vitamin D deficient (<20 ng/mL), eight (17.8%) as insufficient (20-30 ng/mL), and only two (6.6%) had adequate levels (>30 ng/mL) (Figure 1). Notably, 42 out of 45 patients (93.4%) with tibial plateau fractures had either vitamin D deficiency or insufficiency, although vitamin D levels were not statistically significantly different between the fracture and control groups (p = 0.415) (Figure 2). ROC analysis did not identify any individual biochemical marker, including phosphate, ALP, calcium, or 25(OH)D, as a statistically significant predictor of fracture (p > 0.05) (Figure 3). According to the multiple logistic regression model (including age, gender, and BMD as predicting factors), the presence of osteopenia was strongly associated with fracture risk: patients with osteopenia were found to be 32 times more likely to sustain a tibial plateau fracture compared to individuals with normal BMD (Table 2).

**Table 1:** Demographic and clinical characteristics of tibial plateau fracture group.

		Ν	%
Gender	Male	33	73.3
	Female	12	26.7
Schatzker classification	1-2-3	18	40.0
	4-5-6	27	60.0
Mechanism of injury	Low energy	9	20.0
	High energy	36	80.0
Bone mineral density	Normal	31	68.9
	Osteopenia	14	31.1
Age	Mean ± SD (min-max)	48.93 ± 13.28 (21-78)	
	Median( min-max)		
Z-score (age < 50)		0.2 (-1.7 / 0.4)	
T-score (age ≥ 50)		-0.9 (-1.8 / 0.0)	

**Table 2:** ROC analysis of biochemical markers in differentiation between the plateau fracture and the control group. * Higher values denote plateau fracture. † Lower values denote plateau fracture. ROC: receiver operating characteristic; AUC: area under the curve; SE: standard error; CI: confidence interval; ALP: alkaline phosphatase; Ca: calcium; Phos: phosphate.

	AUC	SE	95% CI		p-value
ALP*	0.603	0.065	0.48	0.73	0.140
Ca^†^	0.572	0.068	0.44	0.71	0.302
Phos^†^	0.622	0.065	0.50	0.75	0.081
Vitamin D^†^	0.516	0.068	0.38	0.65	0.821

## Discussion

Tibial plateau fractures are complex injuries of the knee joint, frequently resulting from high-energy trauma in younger individuals but also increasingly observed in older populations due to decreased bone quality and underlying metabolic bone disorders. Although classically associated with road traffic accidents or sports injuries, a considerable proportion of tibial plateau fractures, particularly in elderly or osteopenic individuals, can occur following low-energy trauma, such as simple falls from standing height.

Osteopenia and osteoporosis are well-established risk factors for fragility fractures. In our study, although no participants were found to have frank osteoporosis (T-score or Z- score in participants less than 50 years old ≤ -2.5), a high percentage (14 among 45; 31.1%) of patients with tibial plateau fractures had osteopenia (T-score or Z- score in participants less than 50 years old between -1.0 and -2.5). Importantly, women were significantly more affected than men, and patients who sustained fractures from low-energy mechanisms had a notably higher prevalence of osteopenia (six among nine) than those injured through high-energy trauma (eight among 36) (66.7% vs. 22.2%, p = 0.017). These findings are consistent with prior studies indicating that decreased BMD is a major contributor to fracture risk in both sexes and across age groups [15-17].

Moreover, vitamin D deficiency was highly prevalent in our patient cohort, with 93.4% (43 among 45) of patients exhibiting serum 25(OH)D levels below 30 ng/mL, and 75.6% (34 among 45) below 20 ng/mL. Although no statistically significant association was found between vitamin D levels and the risk of sustaining a tibial plateau fracture in our sample, the overwhelming majority of patients with such fractures were found to have suboptimal vitamin D status. The median value of vitamin D level in our control group was 15.65 ng/ml. Xyda et al. [17], in a recent epidemiological study among 8780 Greeks, found that the mean level of vitamin D was 25.1 ng/mL. A possible explanation for this discrepancy between our results and this study is the small sample of subjects in our control group. Also, our control group consisted mainly of young individuals (mean age = 34 years) compared with the older tibial plateau patients (mean age = 48.93 years, p < 0.005). Additionally, our control group was of urban origin, meanwhile some of the tibial plateau patients were referred to our hospital from rural areas. These findings may show that low vitamin D levels in tibial plateau fracture patients need to be addressed and also highlight the potential role of vitamin D deficiency in impairing bone quality and fracture healing, even if it does not independently predict fracture occurrence in this specific anatomic location [18-20]. Further studies should be done with a larger number of subjects in the control group to confirm that low vitamin D can predict fracture of the tibial plateau.

BMD testing via DEXA remains the gold standard in diagnosing osteoporosis and assessing fracture risk, especially in patients over the age of 50 years or in postmenopausal women. It has been proposed that even patients with low-trauma fractures, regardless of T-score, should be evaluated for underlying bone fragility and managed accordingly [6]. Although our study did not identify statistically significant associations between biochemical markers of secondary osteoporosis (e.g., ALP and phosphate) and tibial plateau fracture risk, this may be attributed to the relatively small sample size. Nevertheless, early identification and correction of metabolic abnormalities, such as vitamin D deficiency, may enhance bone healing and reduce the risk of subsequent fractures [21-23].

Our findings align partially with those of Krause et al. (2018) [12], who demonstrated that osteoporotic patients exhibit reduced bone volume, trabecular connectivity, and subchondral cortical thickness in the tibial plateau, particularly in posterior and central regions. Although we were unable to replicate these microstructural findings due to the lack of histomorphometric or micro-CT data, the observed prevalence of osteopenia and vitamin D deficiency supports the hypothesis that impaired bone quality plays a role in the pathophysiology of tibial plateau fractures.

Limitations of our study include its relatively small sample size, single-center design, absence of long-term follow-up regarding fracture healing or secondary fracture prevention strategies, and the age mismatch between our patients and the control group. Nonetheless, the prospective nature of the study, along with biochemical and radiographic evaluations, provides valuable insights into the metabolic bone status of patients with tibial plateau fractures. Further long-term and age-matched studies should be done to further evaluate our results.

## Conclusions

In summary, our study supports the association between decreased bone quality, particularly osteopenia, and the occurrence of tibial plateau fractures, especially in the context of low-energy trauma. Although vitamin D deficiency was highly prevalent among patients, it did not independently predict fracture occurrence in this study. Future studies with larger sample sizes and long-term follow-up are necessary to validate these findings and to determine the impact of targeted bone health interventions on fracture healing and recurrence.

We propose that patients presenting with tibial plateau fractures, particularly females and those with low-energy mechanisms, should undergo routine assessment of BMD via DEXA, as well as serum vitamin D levels, and also endocrinological evaluation as a standard protocol. In cases requiring surgical management, the orthopedic approach should take into account the potential presence of compromised bone quality, with particular attention to achieving stable fixation in osteoporotic bone.
